# Supplementary material for: Extracellular ATP elicits DORN1-mediated RBOHD phosphorylation to regulate stomatal aperture
Source: Nat Commun. 2017 Dec 22;8:2265. doi: 10.1038/s41467-017-02340-3 (PMC5741621; doi:10.1038/s41467-017-02340-3)
Supplement: Supplementary file 1 — Supplementary Information [file 41467_2017_2340_MOESM1_ESM.pdf]

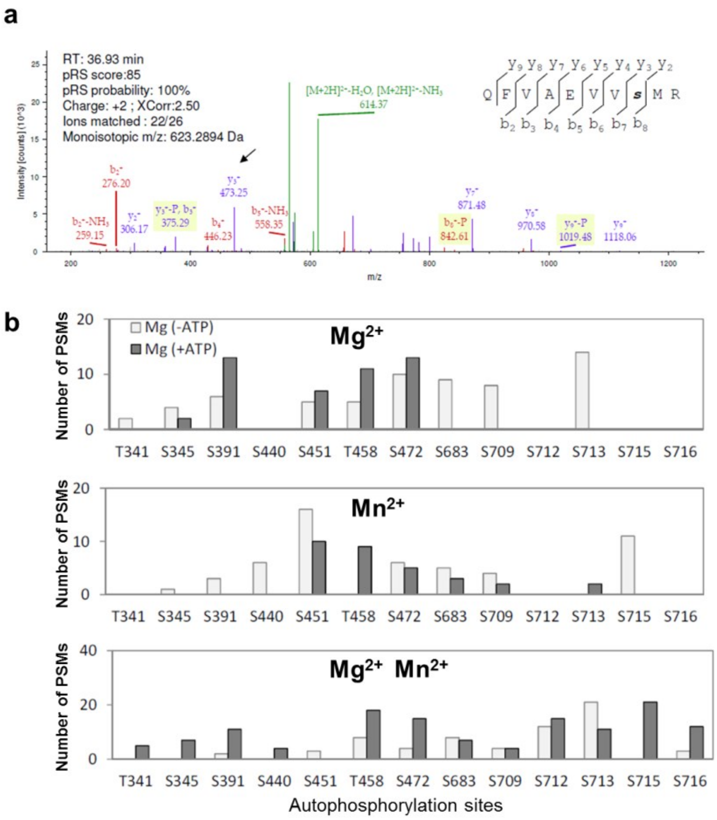

Supplementary Figure 1. Autophosphorylation site mapping of *Arabidopsis* DORN1.

(a) A representative MS/MS spectrum of the phosphopeptide QFVAEVVS(p)MR corresponding to Ser391 of DORN1. Small letters in the sequence (right side) and arrow in the MS/MS spectrum indicates the autophosphorylation site and detection of the phosphorylated residues, respectively. Detailed information of the phosphopeptide identification and detection of phosphorylation site is given on the left corner of the MS/MS spectrum. **(b)** The average phospho peptide spectral matches (PSMs) identified for each phosphosite. ATP, Mg^{2+} and Mn^{2+} ions were present in different PSMs.

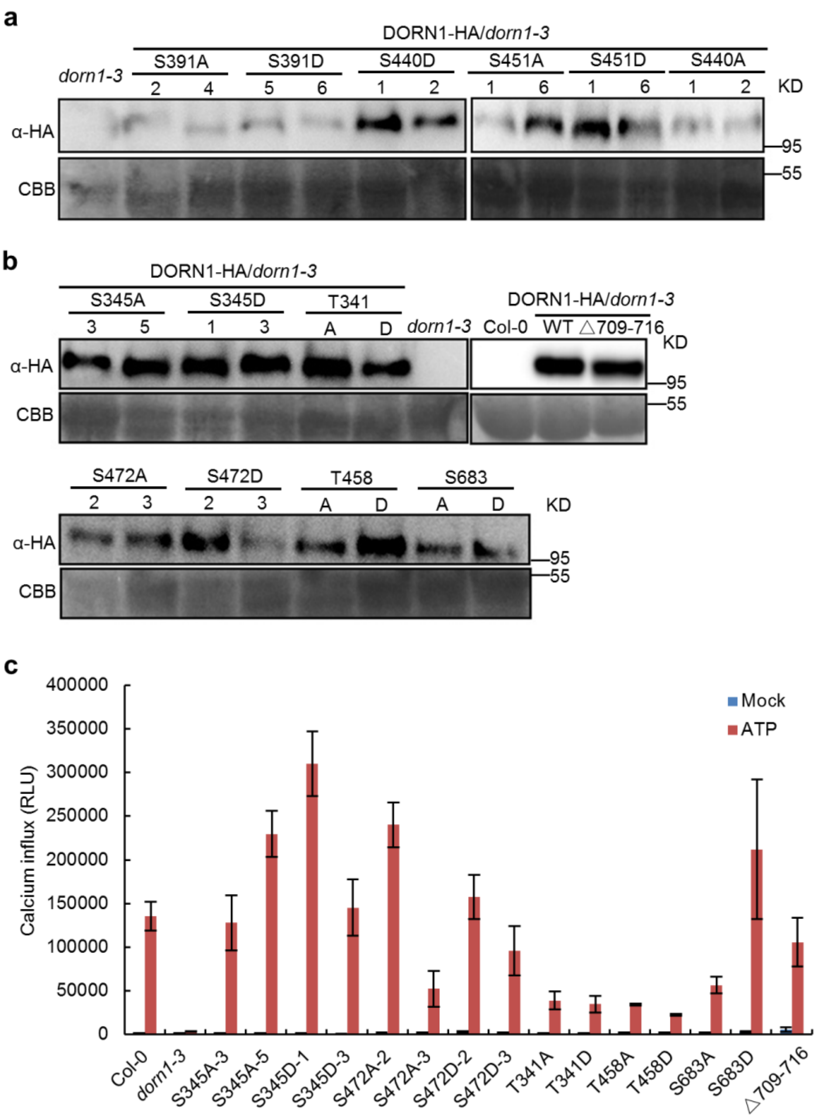

Supplementary Figure 2. Calcium influx and relative expression of different forms of DORN1.

(**a,b**) Relative expression protein levels of *DORN1* in different DORN1 mutant transgenic plants. Total DORN1-HA protein was detected by anti-HA immunoblot. CBB, Coomassie brilliant blue staining. The numbers above the panel indicate different transgenic lines; S, Serine; T, Threonine; A, Alanine; D, Aspartic acid. This experiment was repeated three times with similar results. **(c)** Contribution of different DORN1 autophosphorylation sites other than S391, S440 and S451 to ATP-induced calcium influx. Different transgenic plants were treated with 100 µM ATP and the luminescence was immediately monitored. RLU, relative luminescence units; Error bars indicate ±SEM; n = 8 (biological replicates). △709-716, deletion of amino acids from 709 to 716. This experiment was repeated three times with similar results.

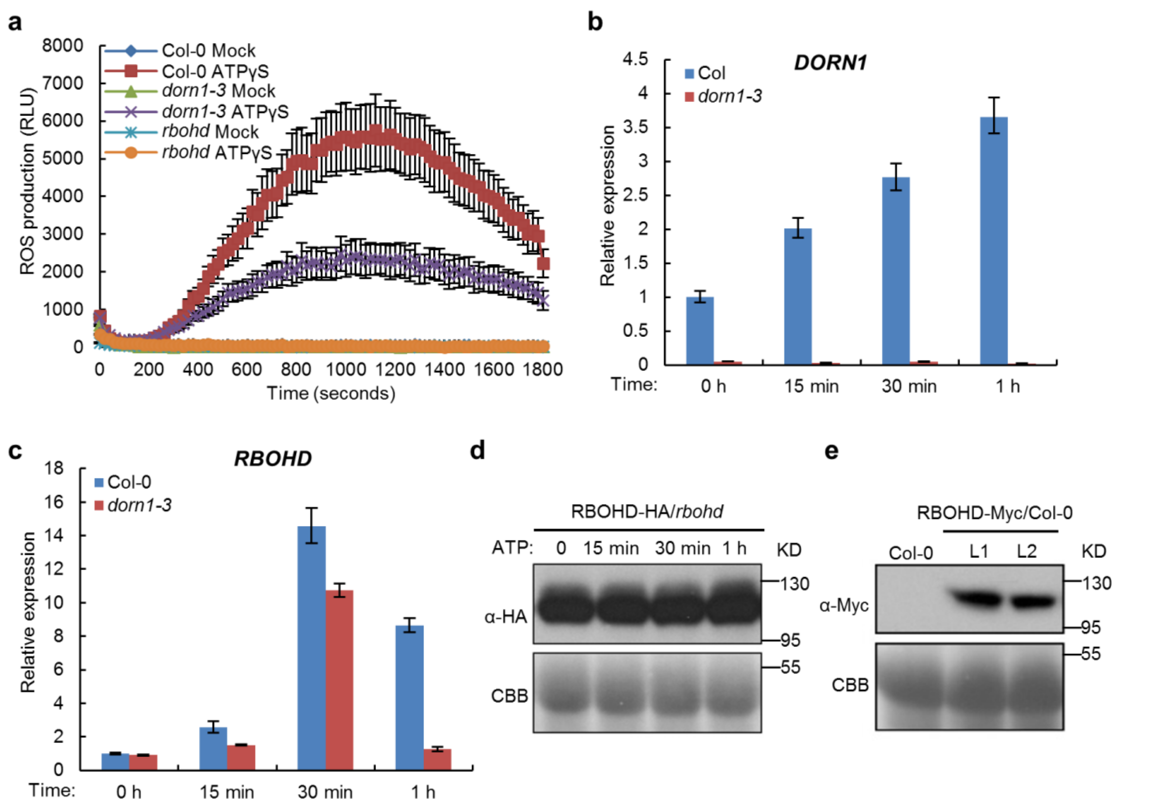

Supplementary Figure 3. ATP induces *DORN1* and *RBOHD* gene expression.

(a) Time-course of eATP-induced ROS production in wild-type, *dorn1-3* and *rbohd* mutants. RLU, relative luminescence units; Values represent the mean ± SEM, n = 8 (biological replicates). This experiment was repeated three times with similar results. **(b, c)** *DORN1* and *RBOHD* expression levels in wild-type and *dorn1-3* mutant seedlings treated with 250 µM ATP. Transcript levels were determined by qRT-PCR and normalized against the expression of ubiquitin, *UBQ*. Bars indicate ± SD, n = 3 (technical replicates). All experiments were repeated three times with similar results. **(d)** RBOHD protein expression level was not altered by ATP treatment. RBOHD protein level was determined by anti-HA immunoblot in *NP::RBOHD×HA/rbohd* expressing transgenic plants after treatment with 200 µM ATP for 0, 15 min, 30 min and 1 h. CBB, Coomassie brilliant blue staining. This experiment was repeated three times with similar results. **(e)** Equal protein expression of CaMV 35S promoter drive RBOHD-Myc in transgenic plants in the Col-0 background. Total RBOHD-Myc protein was detected by anti-Myc immunoblot and CBB staining was used to monitor protein loading. L1 and L2, transgenic line 1 and line 2. This experiment was repeated three times with similar results.

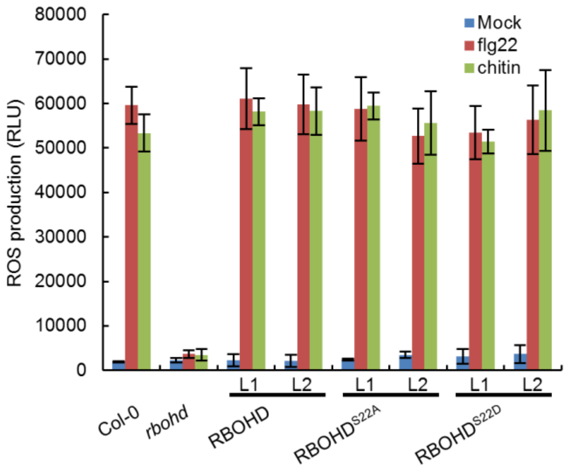

Supplementary Figure 4. S22 does not impair flg22 and chitin-induced RBOHD activation.

ROS production was measured using leaf discs treated with 1 μM flg22 or chitin. RLU, relative luminescence units; Values represent the mean ± SEM, n = 8 (biological replicates). L1 and L2, transgenic line 1 and 2. This experiment was repeated three times with similar results.

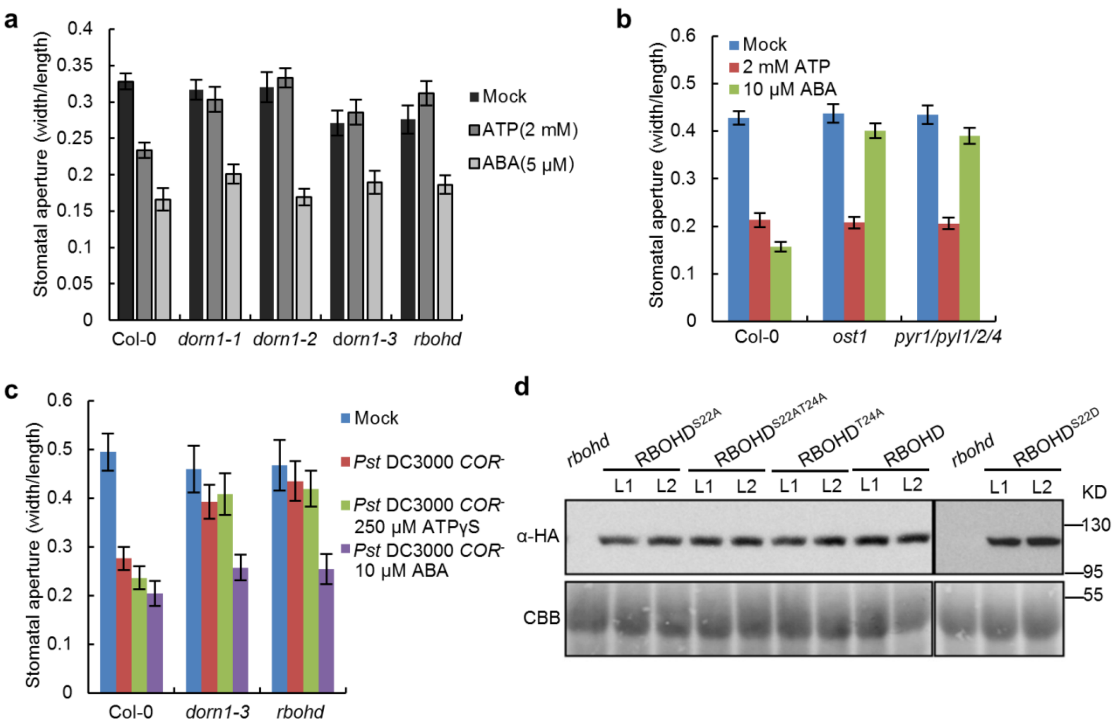

Supplementary Figure 5. DORN1 and RBOHD positively regulate stomatal closure.

(a, b) ATP induced stomatal closure is independent of ABA. The indicated mutant seedlings were treated with 2 mM ATP, 5 μM or 10 μM ABA. The *ost1* and *pyr1/pyl1/pyl2/pyl4* (*pyr1/pyl1/2/4*) quadruple mutants are ABA pathway mutants. Values represent the mean ± SEM, n ≥ 50 (biological replicates). This experiment was repeated three times with similar results. **(c)** The *dorn1-3* and *rbohd* mutants do not close stomata in response to *P. syringae* DC3000 *COR*$^-$ inoculation or treatment with ATP. Stomatal aperture was determined in the leaves of seedlings after treatment with 10^8 cfu/ml (OD_{600} = 0.2) *P. syringae* DC3000 *COR*$^-$, 250 μM ATPγS or 10 μM ABA for 3 h. Values represent the mean ± SEM, n ≥ 50 (biological replicates). This experiment was repeated three times with similar results. **(d)** Equal protein expression of different forms of RBOHD-HA in transgenic plants in the *rbohd* mutant background. L1 and L2, transgenic line 1 and line 2. Total RBOHD-HA protein was detected by anti-HA immunoblot and CBB staining was used to monitor protein loading. This experiment was repeated three times with similar results.

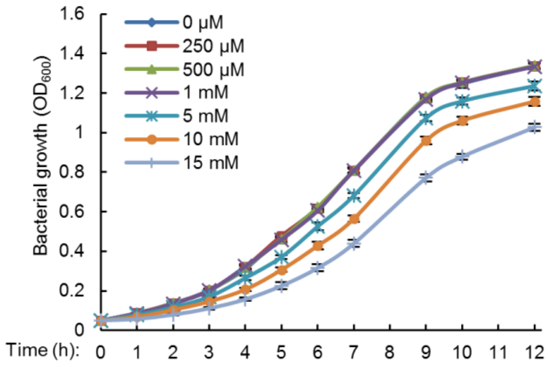

Supplementary Figure 6. Only high concentrations of ATP inhibit bacterial growth.

The growth of *P. syringae* DC3000 in liquid medium at 28℃ over 12 h was monitored by optical density in the presence of increasing concentrations of ATP. Mean ± SD from three independent experiments.

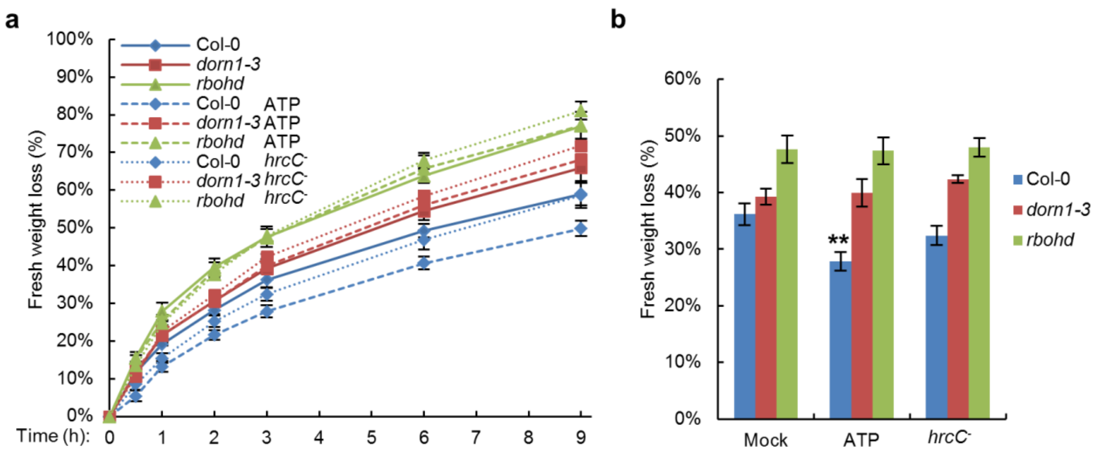

Supplementary Figure 7. DORN1 and RBOHD act together to prevent leaf water loss.

(a) Kinetics of leaves water loss following ATP or bacterial treatment. The leaves of the indicated wild-type and mutant plants were vacuum-infiltrated with 2 mM ATP or a *P. syringae* DC3000 *hrcC*⁻ suspension (OD_{600} = 0.01). **(b)** Fresh weight loss at 3 h shows significantly different values depending on treatment and mutant background. Values represent the mean ± SEM, n = 15 (biological replicates); ***P* < 0.01, Student's *t* test. All above experiments were repeated three times with similar results.

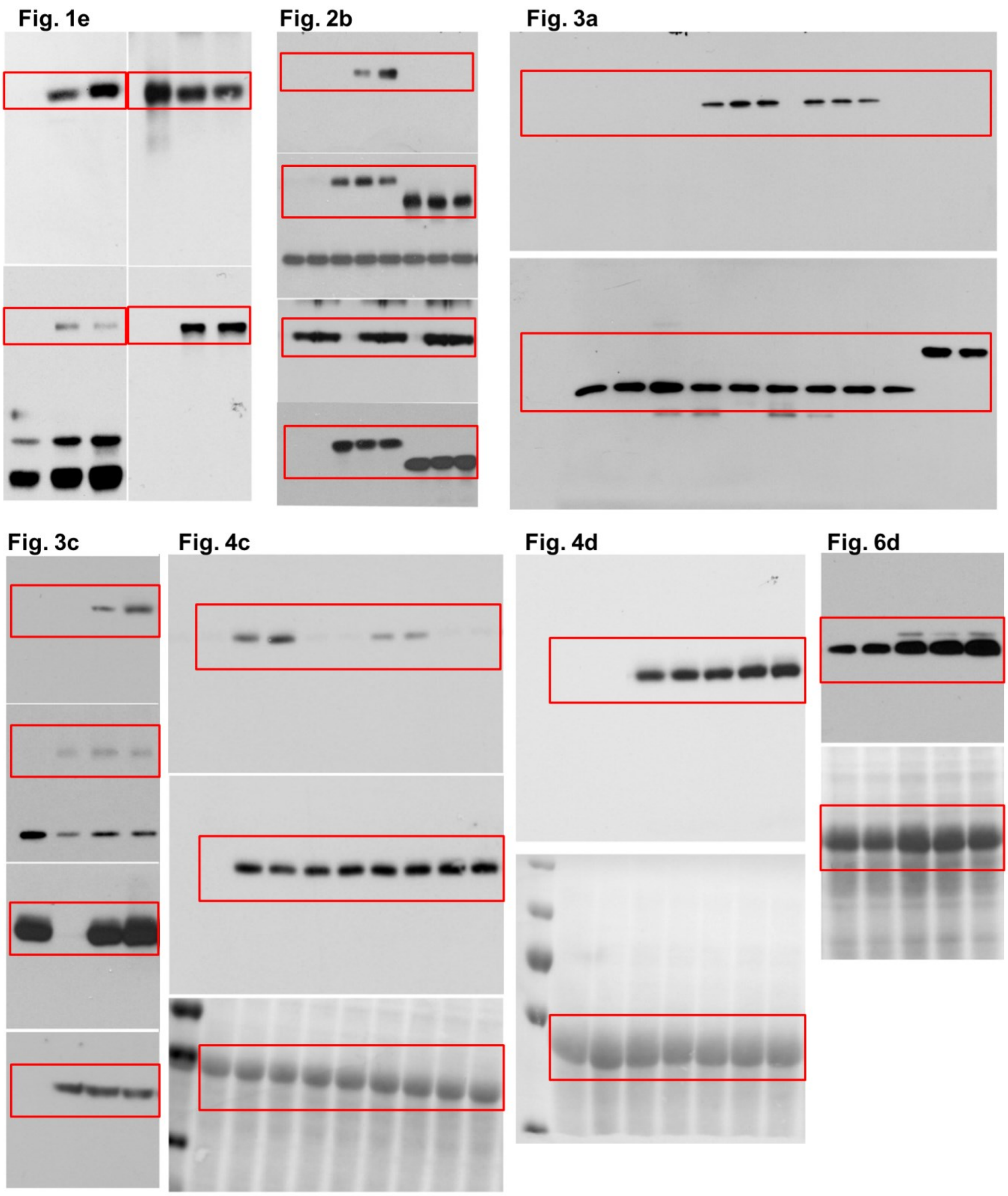

Supplementary Figure 8. Full view data of immunoblots partially shown in main Fig. 1e, Fig. 2b, Fig. 3a, Fig. 3c, Fig. 4c, Fig. 4d, and Fig. 6d.

Supplementary Table 1. ***In vitro*** **autophosphorylation sites of** ***Arabidopsis*** **DORN1**

Phosphopeptide	Position	pRS Score	pRS site probability	MH+ [Da]	XCorr
GFS(p)KDEFLGK	S345	77	S(3): 100.0	1207.54302	2.49
QFVAEVVS(p)MR	S391	144	S(8): 100.0	1245.56902	2.81
ELLLVSEYMPNGSLDEHLFDDQKPVLSWS(p)QR	S440	26	S(13): 0.5;<br>S(27): 20.6;<br>S(29): 78.0	3725.76670	4.92
GIAS(p)ALWYLHTGADQVVLHR	S451	112	S(4): 99.5;<br>Y(8): 0.5;<br>T(11): 0.0	2287.13970	5.60
DVKAS(p)NIMLDAEFHGR	S472	130	S(5): 100.0	1882.85808	5.01
NS(p)LHIVAEPEKPSPAVK	S683	91	S(2): 100.0;<br>S(13): 0.0	1895.97063	3.41
MVTLPAEDPQS(p)NHSSISSQR	S709	67	T(3): 0.0;<br>S(11): 76.7;<br>S(14): 0.3;<br>S(15): 10.7	2280.00371	2.67
MVTLPAEDPQSNHS(p)SISSQR	S712	103	S(14): 92.1;<br>S(15): 6.4;<br>S(17): 0.5	2264.00859	4.48
MVTLPAEDPQSNHSS(p)ISSQR	S713	90	S(15): 85.6;<br>S(17): 0.6;<br>S(18): 0.1	2264.00591	4.55
MVTLPAEDPQSNHSSIS(p)SQR	S715	80	S(15): 8.7;<br>S(17): 82.4;<br>S(18): 8.7	2264.01103	3.69
MVTLPAEDPQSNHSSISS(p)QR	S716	36	S(14): 83.4;<br>S(15): 83.4;<br>S(17): 38.1;<br>S(18): 86.1	2423.94219	2.15
AT(p)KGFSKDEFLGK	T341	47	T(2): 100.0;<br>S(6): 100.0	1587.68816	2.23
GIASALWYLHT(p)GADQVVLHR	T458	116	S(4): 0.0;<br>Y(8): 0.0;<br>T(11): 100.0	2287.13896	5.45
