# Supplementary material for: Extracellular ATP elicits DORN1-mediated RBOHD phosphorylation to regulate stomatal aperture
Source: Nat Commun. 2017 Dec 22;8:2265. doi: 10.1038/s41467-017-02340-3 (PMC5741621; doi:10.1038/s41467-017-02340-3)
Supplement: Supplementary file 3 — Description of Additional Supplementary Files [file 41467_2017_2340_MOESM3_ESM.pdf]

Description of Supplementary Files

File Name: Supplementary Data 1
Description: Details of the autophosphorylation sites of DORN1 using a bottom-up proteomics approach.

File Name: Supplementary Data 2
Description: All the primers used in this study.
